# Who Holds the Plate? Psychotherapists’ Perspectives on Dietary Behavior, Transdiagnostic Evaluation and Interdisciplinary Collaboration in Eating Disorders

**DOI:** 10.3390/nu18071030

**Published:** 2026-03-24

**Authors:** Panagiota Tragantzopoulou, Aikaterini Tragantzopoulou, Vaitsa Giannouli

**Affiliations:** 1 School of Social Sciences, University of Westminster, 115 New Cavendish St., London W1W 6UW, UK; 2 School of Medicine, Aristotle University of Thessaloniki, 54124 Thessaloniki, Greece; atrag@auth.gr (A.T.); giannouliv@hotmail.com (V.G.)

**Keywords:** eating disorders, transdiagnostic evaluation, treatment, interdisciplinary collaboration, dietitians, eating behavior, psychotherapy perspectives, body image, emotion regulation

## Abstract

**Background/Objectives**: Dietary behavior in eating disorders (EDs) is often framed through either nutritional or psychological perspectives, yet emerging evidence suggests that eating may involve a transdiagnostic, emotionally embedded, and relationally negotiated process. While research highlights the role of emotion regulation difficulties, perfectionism, control, and overvaluation of weight and shape in ED maintenance, less is known about how these processes are interpreted and managed in clinical practice across different cultural contexts. This study explored psychotherapists’ perspectives on dietary behavior, nutritional assessment, and interdisciplinary collaboration in ED treatment in Greece and the United Kingdom. **Methods:** Eighteen psychotherapists (9 Greek and 9 British) with experience in treating individuals with EDs participated in in-depth semi-structured interviews. Data were analyzed using reflexive thematic analysis. **Results**: Three themes were developed. First, therapists conceptualized dietary behavior as reflecting broader transdiagnostic psychological processes, particularly perfectionism, control, emotion regulation difficulties, and body image concerns. Second, nutritional assessment and intervention (e.g., food diaries and meal plans) were experienced as emotionally significant practices that required negotiation of timing, meaning, and clients’ readiness for change. Third, interdisciplinary collaboration was described as involving ongoing negotiation of nutritional authority, with therapists balancing nutritional considerations and psychological safety, influenced by contextual differences between UK and Greek mental health systems. **Conclusions**: Findings suggest that dietary behavior in ED treatment may benefit from approaches that integrate psychological and nutritional perspectives. Clinicians may consider attending to clients’ emotional readiness, the symbolic meanings of food, and the dynamics of multidisciplinary collaboration, offering insights that can inform clinical practice and future research.

## 1. Introduction

### 1.1. Eating Disorders as Complex, Chronic, and Transdiagnostic Conditions

Eating disorders (EDs) are severe mental health conditions characterized by persistent disturbances in eating behavior and related cognitions, accompanied by profound physical, psychological, and social impairment [1]. Recognized diagnoses, including anorexia nervosa (AN), bulimia nervosa (BN), and binge-eating disorder (BED), are associated with elevated morbidity and mortality, positioning EDs among the most lethal psychiatric disorders [1]. Global lifetime prevalence estimates range from 0.74 to 2.2% in males and 2.58 to 8.4% in females, with converging evidence that prevalence is increasing across cohorts [2]. Although evidence-based treatments exist, recovery trajectories remain highly variable: approximately 20% of individuals with AN and 10% of those with BN develop a long-standing or severe and enduring illness [3,4,5]. These outcomes signal not only a treatment efficacy problem, but a deeper conceptual challenge, namely, that key aspects of ED psychopathology, particularly dietary behavior, are simultaneously central to most models of illness and yet inconsistently theorized, operationalized, and treated in practice.

Historically, the ED field has been dominated by categorical diagnostic frameworks [6]. However, persistent problems of diagnostic instability, symptom crossover, and high rates of comorbidity have undermined the clinical and theoretical adequacy of diagnosis-specific models, prompting a shift toward transdiagnostic and dimensional approaches. These approaches seek to identify shared psychological mechanisms that cut across diagnostic categories, with the dual aim of improving theoretical coherence and clinical utility.

Fairburn and colleagues’ transdiagnostic model has been especially influential in shaping contemporary understanding of ED psychopathology [7,8]. The model proposes that common maintaining mechanisms operate across ED diagnoses, accounting for shared clinical features, diagnostic migration over time, and the transformation of symptoms (e.g., restrictive AN evolving into BN). At its core is a psychopathology characterized by the overvaluation of weight, shape, and their control, such that self-worth becomes disproportionately contingent on eating- and body-related behaviors [7]. Within this framework, EDs are conceptualized primarily as cognitive disorders, in which maladaptive beliefs and self-schemas drive both behavior and distress [9].

### 1.2. Transdiagnostic Models and the Centrality, Yet Ambiguity, of Dietary Behavior

Dietary behavior occupies a pivotal role within this model. Dietary restraint, rigid food rules, binge eating, and compensatory behaviors are conceptualized not as peripheral symptoms, but as mechanisms through which core psychopathology is enacted and maintained. Restriction and control of eating are understood to temporarily regulate self-esteem, reduce perceived threat, and generate a sense of mastery, while paradoxically intensifying preoccupation with food, weight, and appearance [7,8,9]. These processes are further sustained through behavioral and cognitive mechanisms such as shape and weight checking or avoidance, misinterpretation of bodily sensations as “feeling fat,” social withdrawal, and progressive narrowing of valued life domains.

Beyond eating-specific processes, the transdiagnostic model incorporates broader vulnerabilities drawn from general psychopathology, including clinical perfectionism, low self-esteem, mood intolerance, and interpersonal difficulties [7,8]. These factors do not deterministically cause EDs; however, when present, they interact dynamically with dietary behaviors, amplifying both severity and chronicity. A substantial body of empirical work provides partial but not definitive support for the transdiagnostic model. Structural equation modeling studies demonstrate that low self-esteem, overvaluation of weight and shape, and ED behaviors are similarly linked across AN and BN [10,11]. However, such models require specifying pathways in advance, limiting their capacity to detect unanticipated or more complex relationships among variables.

More recent network-analytic approaches offer a more exploratory lens. Studies applying network analysis to large clinical samples show that overvaluation of shape and weight consistently occupies a central position across ED diagnoses, with strong connections to dietary restraint and low self-esteem [12,13]. Binge eating and compensatory behaviors also form tightly linked clusters. At the same time, findings challenge key assumptions of restraint theory: dietary restraint is not always strongly linked to binge eating, and mood intolerance does not consistently show robust associations with ED behaviors [13]. These results suggest that while transdiagnostic mechanisms are broadly shared, their functional relationships are more heterogeneous and context-dependent than originally proposed.

Dimensional research further complicates categorical assumptions. Studies examining lifetime eating disorder behaviors (LEDBs) indicate that the number and intensity of disordered eating behaviors are more closely related to functional impairment than diagnostic category per se [14]. Importantly, these impairments appear to be shaped primarily by non-shared environmental factors, such as parental conflict, criticism, and weight-related commentary, rather than diagnosis alone. This reinforces the clinical relevance of transdiagnostic approaches while underscoring that meaning, context, and interpersonal history are integral to understanding dietary behavior in EDs. Relatedly, increasing attention has been given to Orthorexia Nervosa (ON), a construct describing a pathological preoccupation with “healthy” or “pure” eating characterized by rigidity, moralization of food, and distress when dietary rules are violated [10]. Although not formally recognized as a diagnostic category, ON has been discussed as overlapping with transdiagnostic processes such as perfectionism, control, and overvaluation of dietary behavior [10]. This construct is relevant to the present study insofar as it highlights how dietary practices may become central to identity, self-evaluation, and emotional regulation beyond traditional diagnostic boundaries.

### 1.3. Emotion Regulation, Affective Pathways, and Eating Behavior

One of the most consistently supported transdiagnostic mechanisms in ED research is difficulty with emotion regulation. Meta-analytic evidence demonstrates medium to large effect sizes for associations between ED psychopathology and both maladaptive emotion regulation strategies (e.g., rumination, suppression, experiential avoidance) and deficits in adaptive strategies (e.g., emotional awareness, clarity, acceptance, problem-solving, and cognitive reappraisal) [15]. These associations are observed across clinical, bariatric, and non-clinical samples, suggesting that emotion regulation difficulties are not merely secondary to EDs but play a substantive role in their development and maintenance [16,17,18,19].

These findings align with the dual-pathway model of eating pathology, which posits that ED symptoms emerge via interacting pathways of unhealthy dieting and negative affect, both driven by sociocultural pressures and internalization of appearance ideals [20,21,22]. Within this framework, dietary restriction exacerbates negative affect through psychological failure experiences and the physiological effects of caloric deprivation, while binge eating functions as a maladaptive attempt to regulate distress. Crucially, either pathway may be sufficient to initiate eating pathology, highlighting that dietary behavior serves multiple psychological functions beyond nutrition alone.

At the intrapsychic level, individuals with EDs frequently describe a highly critical internal “eating disorder voice” that monitors, judges, and regulates eating, body shape, and weight. This voice is often experienced as simultaneously protective and punitive—offering comfort, certainty, or control while exerting intrusive and coercive pressure [23]. The struggle to separate from this internalized voice, coupled with fear of losing its perceived protective function, poses a significant barrier to change and recovery. These findings suggest that disordered eating behaviors are not merely habits to be corrected, but meaningful psychological strategies embedded within systems of self-regulation, identity, and relational experience.

More recent theoretical developments have extended transdiagnostic models by emphasizing metacognitive functioning (i.e., the capacity to monitor, interpret, and regulate one’s own mental states). Researchers propose that individuals with EDs exhibit specific metacognitive impairments, particularly in understanding emotions, differentiating internal states, and responding flexibly to distress [24,25]. These impairments are closely linked to emotion dysregulation and are more pronounced in individuals with histories of childhood adversity [26,27].

Latent profile analyses reveal distinct metacognitive profiles across ED diagnoses, ranging from high-functioning to severely impaired, with the latter associated with elevated depression, emotional dysregulation, trauma exposure, and personality dysfunction [28]. From this perspective, eating behaviors can be understood not only as cognitive or behavioral strategies, but as metacognitive tools (i.e., ways of organizing, simplifying, or avoiding complex internal experiences). This further reinforces the argument that dietary behavior is psychologically meaningful, symbolic, and embedded within broader systems of self-regulation and interpersonal experience.

### 1.4. Dietary Intervention and Interdisciplinary Ambiguity

Despite the centrality of dietary behavior within transdiagnostic, affect-regulation, and metacognitive frameworks, its clinical management remains strikingly under-theorized and inconsistently operationalized. Dietitians are widely recognized as essential members of multidisciplinary ED treatment teams, with established roles in refeeding, nutritional rehabilitation, and the normalization of eating patterns [29,30]. However, there is a notable paucity of empirical evidence guiding the specific content, mechanisms, and effectiveness of dietetic interventions, with most treatment guidelines relying on expert consensus rather than robust outcome data [31,32].

Reviews of treatment manuals further expose this inconsistency. Although the majority include nutritionally focused content, fewer than half explicitly recommend dietitian involvement, suggesting that dietary change is frequently managed within psychologically led treatments without formal interdisciplinary integration [33]. This blurring of professional boundaries is reflected in qualitative research indicating that therapists often hold strong personal and professional beliefs about the importance of diet for mental health and may address nutritional issues within therapy, even when working in isolation from dietitians or without supervisory support [34]. Such practices may contribute to role conflict, theoretical incoherence, and fragmentation of care within multidisciplinary systems.

Outpatient ED treatment is widely recommended to be delivered by multidisciplinary teams comprising mental health professionals, dietitians, and medical practitioners [35,36,37]. In broader healthcare contexts, high-quality interprofessional collaboration is robustly associated with improved patient outcomes, service efficiency, and clinician satisfaction [38]. Yet evidence specific to team-based outpatient ED treatment remains limited and methodologically weak. Scoping and qualitative reviews indicate that while dietitians are commonly included in treatment teams, principles of Interprofessional Collaborative Practice, such as role clarity, shared goals, and coordinated intervention, are inconsistently articulated and often addressed superficially [39]. Emerging research highlights that therapeutic alliance differs meaningfully across patient–dietitian and patient–psychotherapist dyads, with illness severity and general psychopathology shaping these alliances in distinct ways [40]. Qualitative studies in family-based and outpatient contexts further reveal both the benefits and risks of dietetic involvement, including concerns about over-focusing on nutrition, undermining relational processes, or diminishing confidence in other caregivers [41]. Thus, the literature reveals a critical tension: dietary behavior is theoretically central to EDs, yet its clinical management remains fragmented, contested, and insufficiently integrated within psychological treatment frameworks.

Taken together, the existing literature positions dietary behavior at the intersection of transdiagnostic psychopathology, emotion regulation, metacognition, and interdisciplinary care. Yet, despite its theoretical centrality, there remains a significant gap in understanding how therapists themselves conceptualize dietary behavior as an expression of psychological processes, how they assess and intervene with eating in clinical practice, and how they experience and negotiate collaboration with dietitians when addressing dietary change. Most existing research has focused either on psychological mechanisms of EDs or on dietetic practice in isolation, with far less attention to how these domains are integrated -or fail to be integrated- within real-world clinical practice. Consequently, there is limited empirical insight into how therapists make sense of nutrition within treatment, how they balance psychological and dietary priorities, and how interdisciplinary boundaries shape clinical decision-making.

The present study directly addresses these gaps by exploring therapists’ perspectives on dietary behavior, nutritional intervention, and interdisciplinary collaboration in the treatment of disordered eating. In doing so, it seeks to illuminate how transdiagnostic psychological factors are interpreted in relation to eating, how dietary processes are understood within therapeutic work, and how collaboration with dietitians is experienced and negotiated in practice. By foregrounding therapists’ perspectives, this study aims to inform the development of more theoretically coherent, psychologically meaningful, and genuinely collaborative models of eating disorder treatment, models that better align clinical practice with contemporary understandings of EDs as complex, transdiagnostic, and emotionally embedded conditions.

## 2. Materials and Methods

### 2.1. Study Design

This study employed a qualitative research design to explore therapists’ perspectives on dietary behavior, nutritional intervention, and interdisciplinary collaboration in the treatment of disordered eating. The research questions that will be explored are (1) How do therapists understand dietary behavior as an expression of transdiagnostic psychological processes in disordered eating? (2) How do therapists describe the assessment and intervention of dietary behavior in clinical practice, and what psychological meanings are attached to nutritional tools and strategies? And (3) How do therapists experience and negotiate interdisciplinary collaboration with dietitians when addressing dietary change in disordered eating? A qualitative approach was selected because it allows for the in-depth exploration of complex phenomena, capturing the nuanced ways in which therapists conceptualize and enact interventions with clients [42]. Given the exploratory and context-dependent nature of the research questions, a qualitative design was particularly appropriate to uncover therapists’ experiences, beliefs, and reasoning, rather than quantifying their prevalence or testing predefined hypotheses.

### 2.2. Participants and Recruitment

Participants were purposively sampled to include therapists with substantive clinical experience in working with individuals presenting with disordered eating, ensuring that each participant could provide informed and practice-based insights into both the psychological and nutritional dimensions of care. Purposive sampling was selected because the study sought depth of understanding from information-rich cases rather than statistical representativeness [43]. Inclusion criteria were as follows: (1) Holding a recognized professional qualification as a psychologist, psychotherapist, or counselor, accredited by a relevant national professional body in Greece or the United Kingdom; (2) A minimum of two years of post-qualification clinical experience in mental health; (3) Direct experience of working therapeutically with at least five clients presenting with disordered eating behaviors or diagnosed eating disorders within the past three years; and (4) Current or recent (within the last 12 months) clinical practice involving clients with eating difficulties. Exclusion criteria included: (1) Clinicians whose work was limited to brief crisis intervention or assessment-only roles without ongoing therapeutic engagement; and (2) Therapists with no recent clinical contact with clients presenting with eating difficulties.

Recruitment took place between March 2023 and December 2024 through professional networks, national psychological and psychotherapeutic associations, specialist ED services, and snowball sampling via professional referrals. This strategy enabled the inclusion of participants working across a range of clinical contexts (e.g., public services, private practice, outpatient clinics, and specialist ED services), theoretical orientations (e.g., CBT, psychodynamic, systemic, integrative), and levels of experience, thereby enhancing the diversity and credibility of the sample.

A total of 18 therapists participated in the study, with 9 practicing in Greece and 9 in the United Kingdom. Prior to interviews, participants completed a brief demographic and professional questionnaire capturing gender, professional qualification, years of clinical experience, primary therapeutic orientation, current clinical setting, and extent of experience with EDs (see Table 1). This information was used to contextualize the analysis rather than to make quantitative comparisons.

### 2.3. Data Collection

Data were collected through in-depth, semi-structured interviews, chosen for their capacity to elicit rich, nuanced accounts of therapists’ experiences while allowing flexibility to pursue issues raised by participants in real time [44]. This approach was particularly appropriate given the study’s focus on complex, subjective, and context-dependent phenomena such as the psychological meanings of dietary behavior and interdisciplinary clinical decision-making.

All interviews were conducted by the first author, a researcher with a background in psychology and qualitative research and prior clinical familiarity with eating disorder contexts. The interviewer had no prior personal or professional relationship with participants before recruitment. This positioning allowed for informed yet non-assumptive engagement with participants’ accounts.

Interviews were conducted either via secure online video conferencing platforms (e.g., Microsoft Teams) or in person when feasible and preferred by participants. Online interviews facilitated access to therapists across diverse geographic regions within Greece and the UK, while in-person interviews were conducted when participants were local and preferred face-to-face engagement. Each interview lasted between 45 and 75 min and was audio-recorded with participants’ consent and later transcribed verbatim.

Interviews with participants based in Greece were conducted in Greek, while those with participants in the United Kingdom were conducted in English. Greek-language interviews were transcribed in the original language and subsequently translated into English by the first and second authors, who are fluent in both languages. To preserve meaning and nuance, particular attention was paid to culturally specific terms and idiomatic expressions during translation. Where necessary, original Greek excerpts were revisited during analysis to ensure interpretive accuracy, and translated quotations were selected to best reflect participants’ intended meanings.

An interview guide (see Table 2) was developed collaboratively by the research team and refined following two pilot interviews (which were not included in the final dataset). The guide covered four broad domains: (a) Therapists’ conceptualizations of dietary behavior in EDs; (b) Experiences of nutritional assessment and intervention; (c) Interdisciplinary collaboration with dietitians and other professionals; and (d) Perceived cultural and systemic influences on clinical practice in Greece and the UK. The guide was used flexibly, allowing participants to elaborate on issues they considered clinically salient and enabling the interviewer to follow up on emergent themes.

At the start of each interview, participants received a detailed verbal and written explanation of the study’s aims, the voluntary nature of participation, confidentiality arrangements, and their right to withdraw without consequence. Participants provided informed consent prior to data collection. At the end of each interview, participants were debriefed, invited to ask questions, and provided with contact details for the research team should they wish to clarify or add information later.

### 2.4. Data Analysis

Interviews were audio-recorded with participants’ consent and transcribed verbatim by the members of the research team. Transcripts were checked for accuracy against the audio recordings and subsequently anonymized; all identifying information (names, workplaces, and specific locations) was removed to protect participant confidentiality. Data were analyzed using reflexive thematic analysis, following the principles outlined by Braun and Clarke [45]. This approach was selected because it is particularly well suited to examining complex, contextual, and meaning-laden phenomena, such as therapists’ understandings of dietary behavior, emotional processes, and interdisciplinary collaboration in EDs treatment. Rather than seeking to quantify experiences, reflexive thematic analysis enabled a deep, interpretive exploration of how participants constructed meaning around their clinical practices.

The analysis proceeded through iterative and recursive phases rather than a linear coding process. First, three researchers independently familiarized themselves with the data by repeatedly reading the transcripts and making initial reflexive notes. Second, each researcher generated preliminary codes across the dataset, focusing on segments of text relevant to the research questions. Coding was semantic and latent, capturing both explicit content and underlying meanings.

Coding and theme development were conducted across the full dataset rather than separately by national group. While attention was paid to potential cultural and systemic differences between Greek and UK contexts during analysis, themes were developed to reflect shared patterns of meaning as well as points of divergence across the dataset. Where relevant, contextual nuances were retained within themes rather than treated as analytically separate categories.

The research team then met regularly to compare and refine codes, discuss interpretive differences, and develop preliminary themes. Potential themes were reviewed in relation to both coded extracts and the entire dataset to ensure coherence and analytic depth. Themes were subsequently defined and named through collaborative discussion, with attention to their conceptual distinctiveness and relevance to the research aims.

Discrepancies in coding, particularly at the latent level, were not treated as errors requiring consensus, but as opportunities for deeper analytic engagement, consistent with a reflexive thematic analysis approach. When differences in interpretation arose, these were explored through detailed discussion in analytic meetings, where researchers revisited the original data extracts, examined underlying assumptions, and considered multiple possible meanings. Rather than aiming for full agreement, the team worked toward richer, more detailed interpretations that could accommodate complexity within the data. Decisions about coding and theme development were guided by coherence with the dataset as a whole and alignment with the study’s analytic aims, thereby enhancing the robustness and credibility of the analysis.

Data sufficiency was determined through an emphasis on informational power and depth rather than numerical saturation. The research team judged that the sample provided sufficiently rich, detailed, and diverse accounts to support the analytic claims, based on the specificity of the study aims, the quality of dialogue, and the consistency and depth of the themes across participants. Ongoing analysis during data collection indicated that additional interviews were yielding elaborations rather than substantially new thematic patterns, supporting the decision to conclude recruitment at 18 participants.

Throughout the process, reflexive memos were maintained to document analytic decisions, emerging interpretations, and moments of uncertainty or disagreement. This created an audit trail that enhanced analytic transparency and rigor. In addition, regular analytic meetings were held throughout the coding and theme development phases, during which the team systematically reviewed coded extracts, challenged interpretations, and refined thematic boundaries. These discussions formed a core part of the analytic process and contributed to the credibility and coherence of the final thematic structure.

### 2.5. Reflexivity and Researcher Roles

The research team comprised three researchers with backgrounds in psychology, psychotherapy, medicine, and qualitative research. Their differing clinical orientations and epistemological positions were treated as an analytic resource rather than a limitation, enabling multiple interpretive lenses to be applied to the data. Given the interpretive nature of reflexive thematic analysis, the researchers acknowledged that their professional experiences with eating disorders, beliefs about treatment, and cultural perspectives could shape data interpretation. To address this, they engaged in ongoing reflexive practice throughout the study. Reflexivity involved: (1) Maintaining individual reflexive journals documenting assumptions, emotional responses, and analytic insights., (2) Regular team discussions in which researchers critically examined how their own perspectives might influence theme development., and (3) Explicit consideration of how cultural positioning (e.g., UK vs. Greek contexts) might shape both participant accounts and researcher interpretations. This reflexive process aimed to ensure that themes were grounded in participants’ narratives rather than solely reflecting researchers’ preconceptions. The first author, who conducted all interviews and led the initial stages of analysis, engaged in continuous reflexive journaling to critically examine how their dual role as interviewer and analyst, as well as their familiarity with the topic, might shape data generation and interpretation. This reflexive engagement was complemented by collaborative analysis with the wider research team, helping to balance individual perspectives and support interpretive rigor.

### 2.6. Ethical Considerations

Ethical approval for the study was granted by the University of Derby Ethics Committee, where the data were collected, prior to the authors’ subsequent institutional moves. All participants received an information sheet detailing the study’s aims, procedures, potential risks, and their rights as participants. Written informed consent was obtained before each interview, including consent for audio recording and use of anonymized quotations in publications. Participants were informed that participation was voluntary and that they could withdraw at any point without providing a reason and without any professional or personal consequences. Confidentiality was ensured through removal of identifying details from transcripts, use of pseudonyms or participant numbers, and secure, password-protected data storage accessible only to the research team. Given the potentially sensitive nature of discussing clinical experiences with eating disorders, participants were reminded that they could pause or discontinue the interview at any time. At the end of each interview, participants were debriefed and offered the opportunity to ask questions about the study.

## 3. Results

The analysis of the interviews resulted in three main themes (Table 3) that address the main research questions.

### 3.1. Theme 1: Dietary Behavior as an Expression of Transdiagnostic Psychological Processes

Across interviews, therapists consistently described dietary behavior not as a neutral or purely nutritional practice, but as a primary site through which broader psychological processes are enacted and maintained. Rather than viewing food choices in isolation, participants emphasized how eating routines, dietary rules, and food avoidance often reflect transdiagnostic vulnerabilities such as perfectionism, control, emotion regulation difficulties, and body image concerns. In this sense, dietary behavior functioned as both a medium for managing internal distress and a visible manifestation of deeper psychological dynamics. These patterns were described across participants in both Greece and the United Kingdom. While the study was not designed as a formal cross-cultural comparison, therapists’ accounts suggested broadly shared clinical understandings, with cultural nuances emerging at the level of context rather than fundamentally different processes.

#### 3.1.1. Perfectionism and Control Enacted Through Dietary Rules and Routines

Therapists frequently described clients’ dietary practices as highly structured, rule-governed, and inflexible, reflecting underlying perfectionistic standards and a strong need for control. Food rules were often experienced by clients as providing certainty, predictability, and a sense of mastery, particularly in contexts of emotional or interpersonal instability. Participants noted that adherence to dietary routines could become a central organizing principle of daily life, with deviations experienced as deeply distressing.

One UK-based therapist explained:

*“For many of my clients, food becomes the one area where they feel they can get things exactly right. The rules around eating are very precise—what time, what quantity, what combination—and following them gives a sense of control that they don’t feel elsewhere.”*

Similarly, a Greek therapist described how dietary control often extended beyond health goals, becoming a measure of personal discipline and self-worth:

*“It’s not just about eating well. It’s about being disciplined. If they follow the plan perfectly, they feel successful; if they don’t, it’s experienced almost like a personal failure.”*

Therapists emphasized that these patterns were not necessarily driven by nutritional knowledge or health outcomes, but by an internalized demand for flawlessness. Dietary “slips” were commonly associated with harsh self-criticism and compensatory behaviors, reinforcing rigid eating patterns.

As one participant noted:

*“What stands out is how unforgiving they are with themselves around food. One small deviation and the response is punishment—more restriction, more rules. The food itself becomes secondary to the need to stay in control.”*

Across accounts, dietary routines were described as serving a regulatory function, offering psychological containment in the face of uncertainty. However, therapists also highlighted how this reliance on control through food could limit flexibility, increase anxiety, and interfere with adaptive eating and social functioning.

A subtle distinction emerged in how control was described across contexts. UK therapists tended to conceptualize dietary rigidity in terms of cognitive-behavioral processes and maintenance cycles, whereas Greek therapists more often emphasized moral and characterological dimensions, such as discipline and self-evaluation. This suggests that while the function of control may be similar, its meaning may be differently articulated within each clinical-cultural context.

#### 3.1.2. Body Image Concerns Shaping Nutritional Choices and Avoidance

Body image concerns emerged as a significant influence on dietary behavior, shaping not only what clients ate but also what they avoided. Therapists described how fears related to weight, body shape, or bodily appearance frequently informed nutritional choices, even when clients articulated health-focused rationales for their eating patterns.

A UK therapist reflected:

*“Often the language is about health or feeling ‘clean,’ but when you explore it more, there’s a lot of fear about how certain foods will affect their body—how they’ll look, how they’ll feel in their skin.”*

Participants noted that particular food groups—such as carbohydrates, fats, or processed foods—were commonly avoided due to their perceived impact on body shape, bloating, or weight gain. These avoidances were often justified through nutritional discourse, masking deeper body-related anxieties.

A Greek therapist described this process:

*“They may say, ‘I don’t eat this because it’s unhealthy,’ but underneath there’s a strong concern about appearance. It’s about avoiding feeling heavy, swollen, or out of control in their body.”*

Therapists also highlighted how body image distress could narrow dietary repertoires over time, reinforcing cycles of restriction and vigilance. Eating became a site of constant monitoring, with food choices closely tied to anticipated bodily consequences.

As one participant explained:

*“There’s a lot of anticipatory anxiety. ‘If I eat this, my body will change, and I won’t tolerate that.’ So avoidance becomes a way to manage that fear.”*

Across both cultural contexts, therapists emphasized that these body-driven dietary patterns were rarely experienced by clients as purely aesthetic concerns. Instead, they were deeply intertwined with feelings of safety, acceptability, and self-evaluation, positioning food as a key mediator between internal body image distress and everyday eating behavior.

### 3.2. Theme 2: Nutritional Assessment and Dietary Intervention as Psychologically Charged Clinical Practices

Across accounts, therapists consistently described nutritional assessment and dietary intervention as far more than neutral clinical techniques. Practices such as food diaries, meal plans, and dietary monitoring were understood as emotionally charged encounters that often activated shame, anxiety, resistance, and relational dynamics. These interpretations were grounded in therapists’ clinical observations and should be understood as reflective accounts rather than direct reports of client experience. Participants emphasized that dietary work operates at the intersection of physical health, psychological vulnerability, and moral meaning, requiring careful clinical judgment rather than standardized application.

#### 3.2.1. Food Diaries and Dietary Assessment as Emotional and Relational Encounters

Therapists described food diaries as one of the most emotionally evocative components of assessment. Although intended to document eating patterns, diaries were frequently experienced by clients as tools of surveillance and self-evaluation. Several participants noted that the act of recording food intensified self-criticism and heightened fears of external judgment.

A UK-based therapist explained:

*“For many clients, the diary doesn’t feel like information-gathering. It feels like exposure. They’re very aware that someone else will read it and make sense of who they are from it.”*

Similarly, a Greek therapist described how food records often triggered shame and concealment:

*“Clients will say, ‘I didn’t write everything down because I felt embarrassed.’ That embarrassment tells us a lot about their relationship with food and with themselves.”*

Therapists across contexts noted that incomplete or selectively edited diaries were common and should not be interpreted as lack of motivation.

*“When someone avoids writing certain foods, that’s not resistance—it’s communication. It tells you where the fear is.”*

Another UK therapist reflected:

*“Sometimes the diary becomes harsher than the eating itself. The way they judge what they’ve written is often more distressing than what they actually consumed.”*

These accounts highlight how dietary assessment functions as a relational practice, shaping how clients perceive themselves within the therapeutic space. Experiences of shame and exposure were described by therapists in both Greece and the UK, suggesting that these responses may be characteristic of disordered eating rather than culturally specific. However, differences emerged in how this shame was articulated. UK therapists often framed the diary as eliciting evaluative anxiety linked to being “assessed” or “judged,” whereas Greek therapists more frequently emphasized interpersonal embarrassment and reluctance to disclose. This distinction may point to subtle differences in how self-evaluation versus relational exposure are experienced in the therapeutic context.

#### 3.2.2. Meal Plans as Containment, Compliance, and Psychological Risk

Meal planning was widely described as a necessary but complex intervention. Therapists acknowledged its role in providing structure, predictability, and nutritional stabilization, particularly in the early stages of treatment. However, participants also emphasized that meal plans often carried psychological risks when experienced as rigid or externally imposed.

A Greek therapist noted:

*“The plan can feel like safety at first. It tells them exactly what to do. But for some clients, it quickly becomes another standard they have to meet perfectly.”*

Therapists observed that clients with strong perfectionistic tendencies often engaged with meal plans in an inflexible manner, prioritizing adherence over internal cues.

*“They’ll say, ‘I followed the plan exactly,’ but there’s no sense of listening to hunger or pleasure. The plan replaces their own judgment.”*

A UK-based participant reflected on this tension:

*“You want to support regular eating, but you also don’t want to hand them another system of control.”*

Several therapists described gradually loosening meal plans as treatment progressed, shifting from external structure toward collaborative choice.

*“At some point, the work is about tolerating not knowing—choosing food without a script.”*

Some contextual variation was also evident in how meal plans were positioned within therapy. UK therapists more often described working within structured treatment frameworks in which meal planning formed an established component of care, whereas Greek therapists more frequently described needing to negotiate and individualize the introduction of such structures in less standardized service contexts.

#### 3.2.3. Negotiating Nutritional Change in Relation to Emotional Readiness

Therapists emphasized that dietary intervention must be negotiated in relation to emotional readiness rather than implemented according to fixed timelines. Participants described assessing not only what dietary changes were needed, but whether clients had the psychological capacity to tolerate them.

A UK therapist explained:

*“There are moments where nutritionally you know what would help, but emotionally it would be too much. If you push then, you lose the person.”*

Similarly, a Greek therapist noted:

*“Sometimes the priority is helping the client feel safe in the therapy before we touch the food in any meaningful way.”*

Participants described pacing nutritional change carefully, particularly when food functioned as a primary coping mechanism.

*“If food is their main way of managing anxiety, changing it too fast can feel like taking away their lifeline.”*

This led therapists to frame dietary goals collaboratively and flexibly.

*“We talk about what feels possible now, not what would be ideal on paper.”*

A recurring tension across interviews concerned the balance between stabilizing eating behavior and attending to its psychological meaning. While participants agreed that nutritional adequacy was essential for recovery, they cautioned against approaches that reduced eating difficulties to behavioral targets alone.

A UK-based therapist stated:

*“If you only focus on the meals, you miss why the eating disorder is there in the first place.”*

Food was described as carrying symbolic meanings related to control, morality, identity, and self-worth. Removing or altering eating patterns without addressing these meanings was seen as risking displacement rather than resolution.

A Greek therapist reflected:

*“You can change what someone eats, but if you don’t understand what the food represents, something else will take its place.”*

At the same time, therapists acknowledged that delaying nutritional work could perpetuate risk.

*“It’s a constant negotiation—supporting the body while making sense of the mind.”*

### 3.3. Theme 3: Interdisciplinary Work and the Negotiation of Nutritional Authority

Therapists consistently emphasized that effective treatment of disordered eating relies on interdisciplinary collaboration, particularly between psychological and nutritional professionals. However, participants described this collaboration not as a straightforward division of labor, but as an ongoing process of negotiating authority, responsibility, and clinical priorities. Rather than viewing nutritional guidance as fixed or neutral, therapists highlighted how dietary recommendations must be interpreted, paced, and translated within the psychological context of each client.

#### 3.3.1. Interdisciplinary Collaboration as a Process of Negotiating Nutritional Authority

Across interviews, therapists described collaboration with dietitians as essential for addressing nutritional risk, supporting physiological stabilization, and providing clients with credible dietary guidance. At the same time, participants emphasized that interdisciplinary work required continuous negotiation regarding who holds authority over decisions about food and how that authority is exercised in practice.

A UK-based therapist explained:

*“Dietitians bring expertise that we don’t have, and that’s crucial. But the question is always how that expertise is applied with this particular person.”*

Similarly, a Greek therapist noted:

*“Nutrition can’t be separated from psychology in these cases. The knowledge has to be filtered through what the client can tolerate.”*

Therapists described collaborative work as most effective when communication was regular and dialogical, allowing both disciplines to contribute to formulation and planning.

*“When we’re thinking together, the client experiences it as support. When we’re not, it feels like conflicting authorities.”*

Participants also described situations in which they needed to actively interpret or adapt dietary recommendations in order to preserve therapeutic engagement.

*“It’s not about rejecting the dietitian’s input. It’s about translating it so it doesn’t become another source of control or fear.”*

These negotiations were particularly salient when clients had longstanding difficulties with authority, perfectionism, or compliance. UK therapists more often described collaboration as structured and role-defined within multidisciplinary teams, whereas Greek therapists emphasized more fluid, negotiated, and relationship-based forms of collaboration. This appeared to shape how nutritional authority was experienced and managed in practice.

#### 3.3.2. Balancing Nutritional Necessity and Psychological Safety in Interdisciplinary Care

Therapists repeatedly described a central tension between the clinical necessity of nutritional intervention and the need to maintain psychological safety. While dietary change was viewed as essential for recovery, participants emphasized that the timing, pace, and framing of nutritional prescriptions were critical.

A Greek therapist reflected:

*“Sometimes the plan is nutritionally correct but psychologically impossible. If we push it, the client collapses.”*

Similarly, a UK-based participant explained:

*“There are moments where following the meal plan exactly would undo weeks of therapeutic work.”*

Therapists described their role as mediating between nutritional imperatives and emotional readiness, often advocating for gradual change, flexibility, or temporary adaptation of dietary goals.

*“The question is not just ‘What should they eat?’ but ‘What can they manage right now without increasing risk?’”*

Participants also noted that when interdisciplinary communication was limited, clients could feel caught between professionals, leading to confusion or increased rigidity.

*“If the messages aren’t aligned, clients often choose the most restrictive interpretation.”*

These accounts highlighted the importance of shared language and mutual understanding between disciplines, particularly when working with clients whose relationship with food is closely tied to control, morality, or identity.

#### 3.3.3. Structural and Cultural Contexts Shaping Interdisciplinary Practice

Therapists described how broader healthcare structures and cultural contexts shaped interdisciplinary work. UK-based participants often referred to formal multidisciplinary teams, shared protocols, and institutional support for collaboration.

*“In the UK system, interdisciplinary work is expected. There’s infrastructure for it.”*

In contrast, Greek therapists described more fragmented systems, with collaboration often relying on informal networks and personal relationships.

*“Here, collaboration depends on trust and personal contact. It’s not built into the system.”*

These structural differences influenced how nutritional authority was negotiated, how quickly conflicts could be resolved, and how consistently care was coordinated.

*“When collaboration isn’t formalized, misunderstandings can persist longer.”*

These differences were described not simply as contextual background, but as actively shaping clinical decision-making. UK-based therapists’ accounts reflected work within more formalized multidisciplinary systems, where roles and responsibilities were clearer but sometimes experienced as constraining. In contrast, Greek therapists described greater flexibility alongside increased reliance on personal networks, which could allow for individualized care but also introduced variability in collaboration. While these observations are based on therapists’ accounts rather than formal system comparison, they suggest that structural context plays a meaningful role in how interdisciplinary care is enacted.

## 4. Discussion

The present study examined therapists’ perspectives on dietary behavior, nutritional intervention, and interdisciplinary collaboration in the treatment of disordered eating. The findings illuminate how dietary behavior is understood not as a neutral set of eating practices, but as a psychologically meaningful, relationally embedded, and clinically charged domain that reflects broader transdiagnostic processes. The findings highlight the complexity of working with eating in therapy, revealing tensions between nutritional necessity and psychological safety, and underscoring the inherently interdisciplinary nature of effective treatment.

**Figure 1:**
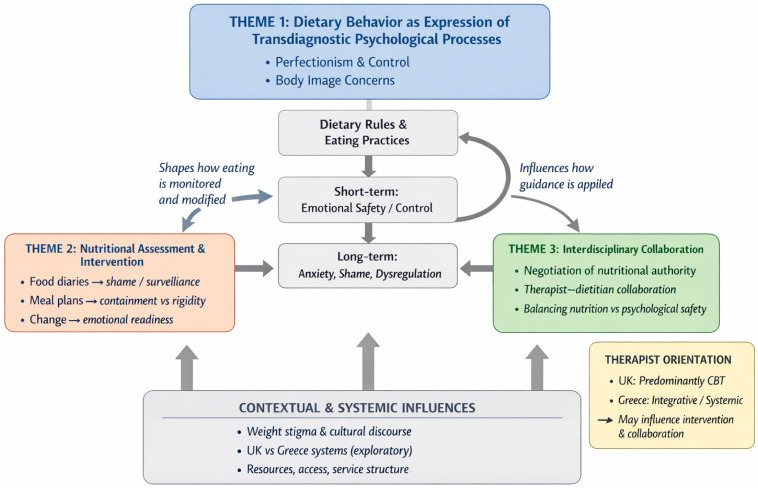
Conceptual map of themes derived from the qualitative analysis The diagram illustrates how dietary behavior (Theme 1), nutritional assessment and intervention (Theme 2), and interdisciplinary collaboration (Theme 3) interact dynamically. Arrows represent relationships described by participants, while contextual and systemic influences (e.g., cultural factors, service structures) and therapist orientation are presented as shaping, rather than determining, these processes.

To enhance clarity and illustrate the relationships between themes, a conceptual map of the findings is presented in Figure 1. The figure visually represents how dietary behavior, nutritional practices, and interdisciplinary processes interact within broader psychological and systemic contexts. While the themes are presented separately for analytic purposes, the diagram highlights their interdependence as described by participants.

### 4.1. Dietary Behavior as a Manifestation of Transdiagnostic Psychological Processes

Therapists consistently framed dietary behavior as an expression of broader psychological dynamics rather than a purely nutritional or behavioral phenomenon. This aligns with transdiagnostic models of EDs, which conceptualize eating behaviors as mechanisms through which core psychopathology, particularly the overvaluation of weight and shape, is enacted and maintained [6,7]. Participants’ accounts suggest that dietary behavior serves functions that extend beyond cognitive distortion or behavioral reinforcement. Eating routines and food rules were described as central organizing structures in clients’ lives, often providing a sense of predictability, containment, and control in contexts of emotional or interpersonal instability. These findings resonate with literature identifying perfectionism and control as key maintaining factors in EDs [6,7], while suggesting that dietary rigidity may also be experienced by therapists as a form of psychological scaffolding rather than merely a maladaptive trait expression.

These observations also resonate with emerging transdiagnostic network models of eating disorders, which identify perfectionism, emotional inhibition, and disrupted interoceptive awareness as central vulnerability nodes linking eating pathology with anxiety and depressive symptoms [46]. Large-scale network analyses suggest that excessive control over bodily states—often enacted through dietary restraint—may be closely intertwined with difficulties in recognizing, tolerating, and integrating internal emotional and bodily signals [46]. From this perspective, rigid eating can be interpreted as not only a symptom but also a core organizing process within a broader transdiagnostic vulnerability network connecting cognitive, affective, and embodied processes.

Therapists described adherence to dietary rules as temporarily reducing anxiety and creating a sense of mastery, while deviations provoked intense self-criticism and compensatory behaviors. This supports the view that restriction and control are deeply entwined with self-worth, positioning food as a moral domain in which personal value is evaluated [6,8]. These dynamics also mirror characteristics described in ON, in which individuals pursue “perfect” eating with excessive rigidity, moralization of food, and intense distress when rules are violated [47]. Although not formally recognized as a distinct diagnosis, ON illustrates how control over food can become an end in itself rather than a means to health [48,49]. Therapists’ descriptions of clients equating dietary adherence with “success” and viewing deviations as “personal failure” suggest that, for some individuals, eating practices may function in ways conceptually consistent with ON, while remaining rooted in broader ED presentations.

From a transdiagnostic cognitive–behavioral perspective, these patterns resonate with Fairburn and colleagues’ model [7,8,9], in which clinical perfectionism, mood intolerance, interpersonal difficulties, and low self-esteem interact with overvaluation of shape and weight to maintain disordered eating. Therapists’ accounts indicate that rigid dietary practices are not diagnosis-specific phenomena but expressions of shared maintaining mechanisms across anorexia nervosa, bulimia nervosa, and binge-eating disorder. In this sense, dietary behavior may be understood, based on therapists’ accounts, as a behavioral interface through which multiple transdiagnostic vulnerabilities converge and are enacted in everyday life.

Therapists also observed that dietary control often provided temporary mastery but ultimately heightened anxiety, which aligns with evidence that excessive self-control may carry affective costs [50,51]. Similarly, attentional hypervigilance toward food-, weight-, and perfection-related cues followed by avoidance may reinforce rigid control and constrain flexible responding [52]. These observations support therapists’ clinical impressions that control-oriented eating may feel protective in the short term but maintain dysregulation over time.

Body image concerns were a pervasive influence on dietary decision-making, shaping both what clients consumed and avoided. Therapists noted that these concerns were often framed in health-oriented language, reflecting the broader cultural convergence of “wellness” discourse with disordered eating [11,12]. Rather than purely visual dissatisfaction, body image distress appeared closely linked to embodied anxiety about how food would affect the body—e.g., feelings of heaviness, bloating, or loss of control. Recent qualitative research similarly highlights the interplay of social media, relational feedback, and internalized beauty ideals in shaping body dissatisfaction and extreme compensatory behaviors [51,53,54]. Therapists’ accounts suggest that clients’ restrictive behaviors may also function as strategies to manage anticipated social judgment and perceived moral responsibility regarding food.

Additionally, weight stigma and social pressures were raised as contextual factors shaping dietary patterns. Experiences of discrimination, family criticism, or societal marginalization may contribute to chronic self-surveillance, shame, and hypervigilance around eating [55]. Therapists’ narratives suggested that clients’ framing of food avoidance as “health” may serve as a defensive strategy within a culture that scrutinizes both bodies and eating practices.

Overall, our findings indicate that dietary behavior functions as a site where internal psychological vulnerabilities intersect with external cultural pressures, stigma, and moralized health discourse. Eating is not merely something that individuals with EDs do; it is a central arena through which safety, identity, control, and social belonging are negotiated [6,7,8,49]. These accounts closely map onto contemporary transdiagnostic frameworks, highlighting the integration of cognitive, emotional, and embodied regulatory processes in shaping eating behavior.

### 4.2. Nutritional Assessment and Intervention as Emotionally Charged Clinical Practices

Therapists portrayed nutritional assessment and intervention as inherently relational and affectively laden rather than neutral clinical procedures. Food diaries, in particular, were described as emotionally evocative artifacts that often intensified shame, self-surveillance, and fear of judgment. Although designed to capture eating patterns, diaries were frequently experienced by clients as forms of exposure rather than information-gathering. Importantly, therapists interpreted incomplete or edited diaries not as resistance but as meaningful communication about where anxiety, shame, or vulnerability was most acute. These accounts suggest that strictly behavioral interpretations of dietary monitoring may overlook the ways in which assessment practices can inadvertently reproduce dynamics of self-policing, perfectionism, and internalized surveillance that may sustain disordered eating, consistent with transdiagnostic formulations of ED maintenance [6,7]. These interpretations are grounded in therapists’ accounts and highlight considerations for approaching dietary assessment with attention to its emotional and relational impact.

These tensions are also reflected in emerging research on digital dietary monitoring tools. A qualitative study of the Recovery Record app shows that self-monitoring technologies can be experienced as either supportive or obstructive depending on how they are used and interpreted [56]. While some patients experience clinician monitoring as fostering connection and accountability, others perceive it as surveillance, exacerbating anxiety and prompting avoidance. This suggests that, from the perspective of therapists and clients, the emotional meaning of monitoring, rather than the method itself, may influence whether it facilitates or undermines recovery. Similarly, feasibility studies comparing food diaries, photographic records, and weighed records in vulnerable populations indicate that all methods carry risks of underreporting and quality deficits, reinforcing therapists’ concerns that “objective” dietary data are always shaped by psychological and relational factors [57].

Meal planning was similarly described as both necessary and potentially problematic. Therapists acknowledged its role in restoring regular eating and physiological stability, particularly in early treatment [27,28]. However, they also noted that meal plans could be co-opted by perfectionistic tendencies, becoming another rigid standard to be met rather than a supportive guide. For some clients, external structure replaced internal attunement to hunger, satiety, and preference, reinforcing control rather than flexibility. Participants therefore emphasized that gradually loosening meal plans over time, shifting from prescriptive structure toward collaborative choice and tolerance of uncertainty, may be particularly helpful. This ambivalence is echoed in recent comparative research on meal planning approaches in weight restoration. Evidence suggests that both calorie counting and plate-by-plate methods can be effective, yet they carry different psychological implications [58]. Calorie counting may promote faster weight gain but could become overly prescriptive or triggering, whereas the plate-by-plate approach may align more closely with intuitive decision-making and relational feeding, particularly within family-based treatment. Therapists’ emphasis on flexibility and gradual autonomy indicates that the most effective nutritional intervention may vary depending on how it interacts with control, anxiety, and caregiver or patient self-efficacy.

Beyond planning, the emotional intensity of actual mealtimes further illustrates why nutritional intervention cannot be treated as purely technical. Systematic reviews indicate that meal support is often necessary because eating itself is a period of heightened distress, during which avoidance, distraction, or concealment frequently emerge [59]. Likewise, meal preparation interventions appear to provide meaningful experiential learning that supports transition to lower levels of care, suggesting that hands-on engagement with food may foster competence, agency, and embodied confidence that written plans alone cannot provide [60].

A recurring theme across interviews was the need to align nutritional change with clients’ emotional readiness. Therapists resisted purely protocol-driven timelines, arguing that pushing dietary change too quickly, especially when food served as a primary coping mechanism, could destabilize clients or undermine therapeutic engagement. This aligns with evidence that eating behaviors often function as maladaptive emotion regulation strategies [14,15,16,17,18], meaning abrupt changes may feel like the removal of a primary coping mechanism. These accounts point to a clinical tension: the necessity of nutritional restoration must be balanced against the potential psychological risks of removing a key regulatory strategy too abruptly.

This emphasis on timing and readiness is consistent with research demonstrating that readiness to change is a strong predictor of treatment outcome in AN, even beyond baseline symptom severity [61]. Gains in motivation and confidence following psychoeducation further suggest that nutritional intervention is most effective when paired with psychological preparation and empowerment [62]. These findings align with the Transtheoretical Model of Change, which frames recovery as a staged process (precontemplation, contemplation, preparation, action, maintenance) rather than a linear progression [63,64]. Therapists’ clinical intuition that dietary change should match a client’s motivational stage reflects these frameworks and underscores the value of tailoring interventions to individual readiness.

Overall, these findings indicate that, in therapists’ experience, nutritional assessment and intervention are not merely adjuncts to psychological treatment but constitute deeply psychological practices. They operate at the intersection of physiology, emotion, relationships, power, and culture, requiring clinicians to navigate not only what clients eat, but how, why, and when change is introduced.

### 4.3. Interdisciplinary Collaboration and the Negotiation of Nutritional Authority

The findings suggest that interdisciplinary work in ED treatment is not simply a matter of dividing responsibilities between therapists and dietitians, but may involve an ongoing process of negotiating authority, timing, and meaning. Therapists valued dietitians’ expertise in nutritional risk and rehabilitation, yet emphasized that dietary recommendations often need to be interpreted and adapted within each client’s psychological context. Rather than presenting nutritional expertise as a straightforward linear transfer from dietitian to therapist to client, therapists described it as relational, contingent, and co-constructed within the therapeutic system. This perspective aligns with broader understandings of nutritional rehabilitation as a cornerstone of ED treatment, particularly for adolescents, where dietitians play a dual medical and psychosocial role in restoring regular eating, addressing physiological risk, and supporting development [65,66]. However, these findings indicate that the perceived effectiveness of dietetic input may depend less on its technical accuracy alone and more on how it is integrated into a psychologically coherent treatment narrative. In this sense, therapists described acting as mediators of nutritional authority, translating dietetic input into forms that clients could tolerate emotionally and meaningfully engage with. This translation work reflects one way in which interdisciplinary collaboration can support recovery, emphasizing the need to align physical restoration with psychological safety.

Rather than viewing nutritional guidance as fixed or universally applicable, participants described their role as translating dietetic input into forms that clients could tolerate emotionally. This was particularly important for individuals with histories of perfectionism, authority sensitivity, or compliance-based relationships with food, vulnerabilities highlighted in transdiagnostic models [6,7]. These accounts suggest that dietetic authority is not simply challenged by clients with EDs, but may be actively reshaped through therapeutic practice to avoid reinforcing rigid, punitive, or rule-bound relationships with food. This aligns with evidence that fostering a sense of autonomy and agency within structured nutritional frameworks can enhance adherence and potentially support long-term recovery [67], highlighting that “authority” in nutritional treatment may need to be balanced with collaboration and empowerment.

Therapists also highlighted a persistent tension between nutritional necessity and psychological safety. While dietary change was viewed as important for recovery, participants frequently described mediating between what was nutritionally ideal and what was psychologically manageable. Misalignment between professionals could potentially exacerbate this tension, with clients often gravitating toward the most restrictive interpretation of conflicting messages. These observations illustrate one way in which interdisciplinary disagreements might inadvertently reinforce tendencies toward control, rigidity, and moralized eating. These concerns mirror broader critiques in the literature about inconsistent interdisciplinary practice and the lack of clearly defined roles within ED teams [31,37]. Systematic reviews on the inclusion of dietitians in outpatient ED treatment further contextualize this tension. While dietetic input consistently improves weight and nutritional intake, its impact on broader psychopathology is more mixed, partly due to variability in how dietetic roles are defined and implemented across services [31]. Such variability may contribute to the negotiation of authority observed in our findings, as unclear professional boundaries could complicate decision-making in complex cases.

Structural and cultural factors appear to influence how interdisciplinary collaboration unfolded. UK-based therapists described more formalized systems of multidisciplinary working, whereas Greek therapists reported reliance on informal networks and personal relationships. These observations should be interpreted cautiously, as the study was not designed as a formal comparative analysis, but they suggest possible contextual influences on clinical practice. These differences could reflect broader systemic disparities rather than individual professional preferences. In the UK, MDT collaboration is embedded within NHS commissioning structures and NICE guidelines, which institutionalize shared responsibility for physical, psychological, and nutritional care [68]. This structure may create clearer role delineation but could also introduce bureaucratic constraints that limit flexibility or responsiveness to individual client needs.

In contrast, the Greek mental health system, particularly for children and adolescents, remains fragmented, under-resourced, and unevenly distributed, with significant reliance on private providers and weak coordination across services [69]. The absence of standardized pathways, limited public specialist provision, and lack of systematic monitoring may intensify the burden on individual clinicians to “make collaboration work” through personal networks rather than formal structures [69]. Within this context, negotiation of nutritional authority becomes not only a clinical issue but may also be shaped by systemic factors, including resource scarcity, access barriers, and service gaps. These dynamics must be understood within a broader sociocultural context, particularly in Greece, where stigma surrounding eating disorders remains pervasive and may complicate help-seeking, professional collaboration, and public investment in services [70]. Such stigmatizing views could marginalize ED care, reduce interprofessional prioritization, and reinforce fragmented treatment pathways, potentially intensifying the challenges therapists described.

Despite these contextual differences, therapists across both countries identified similar challenges in integrating nutritional and psychological perspectives in a coherent and client-centered manner, echoing existing critiques of fragmented care in ED services [32,37]. This convergence suggests that tensions around authority, timing, and meaning in nutritional treatment may reflect intrinsic features of ED care rather than being solely attributable to local systems. The findings also illustrate that interdisciplinary collaboration in ED treatment can involve complex relational, cultural, and systemic negotiation, where nutritional authority is not simply held by dietitians and applied by therapists, but is continuously interpreted, mediated, and reshaped within clinical relationships, institutional structures, and national health systems.

### 4.4. Strengths and Limitations

A major strength of this study is its use of an in-depth qualitative design, which enabled a nuanced exploration of therapists’ lived clinical experiences rather than relying on predefined survey categories or manualized frameworks. By employing reflexive thematic analysis, the study captured the complexity, ambiguity, and affective dimensions of working with dietary behavior in real-world clinical practice—an area that remains relatively under-theorized within ED research, which has historically prioritized symptom-focused or outcome-based approaches [29,30]. This interpretive approach allowed for the identification of relational, emotional, and contextual processes that are often obscured in more standardized methodologies. The inclusion of therapists from two distinct cultural and healthcare contexts (the UK and Greece) is a further strength. This design enriched the analysis by illuminating how structural, institutional, and systemic factors may shape interdisciplinary practice, while also highlighting shared clinical dilemmas that transcend national boundaries [32,37]. At the same time, the study was not designed as a formal comparative analysis, and therefore these cross-contextual insights should be interpreted as exploratory rather than definitive. Rather than assuming cultural equivalence, the study was able to identify both convergences and contextual divergences in how therapists negotiate nutritional authority, emotional readiness, and interdisciplinary collaboration. Additionally, the study contributes to an underexplored area by centering psychotherapists’ conceptualizations of dietary behavior, rather than treating nutrition as solely a medical or dietetic domain [31]. This helps to bridge psychological and nutritional perspectives and advances a more integrated understanding of eating behavior within ED treatment.

The study is limited by its exclusive focus on therapists’ perspectives. The absence of client and dietitian voices means that the findings do not capture how interdisciplinary dynamics, nutritional interventions, or assessment practices are experienced across all stakeholders involved in treatment [37]. Therapists’ accounts may also reflect their professional training, theoretical orientation, or personal clinical philosophies, which were not systematically controlled for in the analysis. In particular, there were notable differences in theoretical orientation across the two national groups, with UK-based therapists more commonly identifying with cognitive-behavioral approaches, while Greek therapists represented a broader mix of integrative, psychodynamic, and systemic orientations. This variation may have influenced how participants conceptualized dietary behavior, control, and intervention. For example, CBT-oriented therapists may be more likely to frame eating behaviors in terms of cognitive and behavioral maintenance mechanisms, whereas integrative or systemic practitioners may place greater emphasis on relational, contextual, or meaning-based processes. As a result, some of the themes identified in the analysis may reflect not only shared clinical experiences but also underlying theoretical frameworks. Given the sample size, it was not possible to systematically examine how orientation shaped responses, and this represents an important area for future research. As a qualitative study with a purposive sample, the findings are not intended to be statistically generalizable. Instead, they offer conceptual and clinically relevant insights that require further empirical examination across broader and more diverse samples [13]. Finally, although cross-cultural differences between the UK and Greece were discussed, the study was not designed as a formal comparative analysis of healthcare systems. Consequently, interpretations of structural differences should be understood as exploratory rather than definitive [32].

### 4.5. Clinical Implications and Future Research

The findings offer a number of tentative implications for clinical practice in ED treatment. First, dietary assessment (e.g., food diaries, monitoring, or apps) may be usefully conceptualized as a relational and emotional process rather than a neutral data-gathering exercise, consistent with evidence that eating behaviors are deeply embedded within emotion regulation systems [14,15,16,17,18]. Clinicians may benefit from remaining attentive to how assessment tools could inadvertently reinforce shame, self-surveillance, or perfectionism.

Second, meal planning may be most helpful when used flexibly and collaboratively rather than rigidly, aligning with transdiagnostic models that caution against rule-based or overly prescriptive eating frameworks [6,7,8]. Participants’ accounts suggest that gradual transitions from structured to autonomous eating can support psychological safety and internal attunement.

Third, therapists may need to carefully consider clients’ emotional readiness when pacing nutritional change, particularly given that disordered eating behaviors often function as maladaptive emotion regulation strategies [14,15,16,17,18]. In this context, abrupt dietary changes could risk destabilizing clients if not psychologically supported.

Fourth, effective interdisciplinary collaboration may extend beyond simple role division toward shared case formulation and ongoing dialogue between therapists and dietitians. This reflects participants’ emphasis on addressing longstanding critiques of fragmented care within ED services [31,37].

Finally, clinicians may need to remain mindful of power dynamics in nutritional guidance, particularly for clients with perfectionistic or control-based eating patterns, where authority-based approaches could risk reinforcing pathology [6,7].

In addition, the findings have potential implications for postgraduate training. Given participants’ accounts that therapists often act as mediators of nutritional authority, particularly in early career stages, training programs may benefit from including explicit teaching on how to negotiate and communicate nutritional guidance within interdisciplinary contexts. This could include developing skills in collaborative formulation with dietitians, managing tensions between nutritional risk and psychological readiness, and reflecting on how clinicians’ own beliefs about food, health, and control may shape their practice. Such content may be particularly relevant for novice therapists, who may have less confidence in navigating these complex dynamics [71,72,73].

Future studies should incorporate the perspectives of both clients and dietitians to develop a more comprehensive understanding of interdisciplinary dynamics in ED treatment and to triangulate therapists’ interpretations [37]. Longitudinal research is needed to examine how therapists’ approaches to nutritional intervention evolve over time and how these shifts relate to treatment outcomes, therapeutic alliance, and clients’ emotion regulation capacities [14,15,16,17,18]. Quantitative or mixed-methods studies could test whether patterns identified in this study—such as heightened shame around food diaries, rigidity in meal plan adherence, or therapist-dietitian misalignment—are associated with treatment engagement, dropout, or recovery trajectories. Finally, comparative research across different healthcare systems would help clarify how structural factors, resource availability, and cultural attitudes toward eating disorders shape interdisciplinary collaboration and clinical decision-making in ED care [32,37].

## 5. Conclusions

This study suggests that dietary behavior in EDs cannot be fully understood—or treated—through purely nutritional or purely psychological lenses. Instead, eating may be more usefully conceptualized as a transdiagnostic, emotionally embedded, and relationally negotiated process that lies at the core of both psychopathology and clinical practice.

By foregrounding therapists’ perspectives, this research offers an exploratory contribution toward a more integrated and psychologically attuned framework for addressing eating in treatment. The findings highlight the complex clinical demands placed on therapists, who must balance nutritional necessity with psychological safety while remaining reflexive about their own potential biases around food, health, and body weight [71,72,73]. This challenge is particularly salient for novice clinicians, who may feel pressured to prioritize either risk management or therapeutic alliance, sometimes at the expense of the other [74,75].

Moreover, the contemporary digital environment—where clients are exposed to conflicting online dietary messages and harmful content—can create additional barriers to treatment engagement and consistency [76,77]. This may further underscore the importance of coordinated multidisciplinary working, which can provide clearer, more consistent, and more supportive guidance for clients.

Given the qualitative design and relatively small sample of therapists, these conclusions should be interpreted cautiously and as indicative rather than definitive. Overall, the study indicates that improving eating disorder treatment may require not only better techniques, but also a more nuanced understanding of dietary behavior as simultaneously biological, psychological, and social—an approach that may support both physical recovery and meaningful psychological change.

## Figures and Tables

**Table 1:** Participant Demographic and Professional Characteristics (N = 18).

Participant	Country	Gender	Professional Qualification	Primary Theoretical Orientation	Years of Clinical Experience	Primary Work Setting	Experience with Eating Disorders	Regular Collaboration with Dietitian (Y/N)
**P1**	Greece	Female	Clinical Psychologist	Integrative	6–10	Private practice	Regular	Yes
**P2**	Greece	Female	Psychotherapist	Integrative	11–15	Private practice	Regular	Yes
**P3**	Greece	Female	Counseling Psychologist	Integrative	2–5	Outpatient clinic	Occasional	Sometimes
**P4**	Greece	Female	Psychotherapist	Integrative	6–10	Private practice	Regular	Yes
**P5**	Greece	Female	Clinical Psychologist	CBT	6–10	Private practice	Regular	Yes
**P6**	Greece	Male	Psychotherapist	CBT	11–15	Outpatient clinic	Regular	Yes
**P7**	Greece	Male	Counselor	CBT	2–5	Community service	Occasional	Sometimes
**P8**	Greece	Male	Family/Systemic Therapist	Systemic	11–15	Family therapy service	Regular	Yes
**P9**	Greece	Male	Counselor	Person-Centered	6–10	Private practice	Occasional	Sometimes
**P10**	United Kingdom	Male	Clinical Psychologist	CBT	11–15	Specialist ED service (non-NHS)	Primary focus	Yes
**P11**	United Kingdom	Male	CBT Therapist	CBT	6–10	Outpatient clinic	Regular	Yes
**P12**	United Kingdom	Male	Counseling Psychologist	CBT	11–15	Private practice	Regular	Yes
**P13**	United Kingdom	Male	Psychotherapist	CBT	6–10	Specialist ED service (non-NHS)	Primary focus	Yes
**P14**	United Kingdom	Male	Clinical Psychologist	CBT	16+	Outpatient clinic	Primary focus	Yes
**P15**	United Kingdom	Male	Psychotherapist	Psychodynamic	11–15	Private practice	Regular	Sometimes
**P16**	United Kingdom	Female	Family/Systemic Therapist	Systemic	11–15	Family therapy service	Regular	Yes
**P17**	United Kingdom	Female	CBT Therapist	CBT	2–5	Community clinic	Occasional	Sometimes
**P18**	United Kingdom	Female	Clinical Psychologist	CBT	6–10	Specialist ED service (non-NHS)	Primary focus	Yes

**Table 2:** Interview Guide.

Section	Topic	Example Questions
**1**	Conceptualization of Disordered Eating and Dietary Behavior	How do transdiagnostic factors influence eating behaviors? How do beliefs about “healthy eating” impact clients’ well-being?
**2**	Assessment and Intervention	How do you assess dietary behavior? How do you use tools like food diaries or meal plans? How do you adapt guidance based on psychological readiness?
**3**	Interdisciplinary Collaboration	How do you collaborate with dietitians? How are decisions about diet negotiated? What challenges arise?
**4**	Contextual and Cross-Cultural Factors	How does your healthcare system influence interventions? How do cultural norms around food affect therapy?
**5**	Closing	Is there anything else important to understand? Any reflections on practice improvement?

**Table 3:** Overview of themes and subthemes derived from the qualitative analysis.

Theme	Analytic Focus	Subthemes
Theme 1. Dietary Behavior as an Expression of Transdiagnostic Psychological Processes	How eating practices reflect and maintain underlying psychological vulnerabilities	1.1 Perfectionism and control enacted through dietary rules and routines 1.2 Body image concerns shaping nutritional choices and avoidance
Theme 2. Nutritional Assessment and Dietary Intervention as Psychologically Charged Clinical Practices	How dietary assessment and intervention are experienced and negotiated in therapy	2.1 Food diaries and dietary assessment as emotional and relational encounters 2.2 Meal plans as containment and constraint 2.3 Negotiating nutritional change in relation to emotional readiness
Theme 3. Interdisciplinary Work and the Negotiation of Nutritional Authority	How therapists collaborate with dietitians and balance nutritional and psychological priorities	3.1 Interdisciplinary collaboration as a process of negotiating nutritional authority 3.2 Balancing nutritional necessity and psychological safety in interdisciplinary care

## Data Availability

Data can be made available upon reasonable request due to restrictions ethical reasons.

## Abbreviations

The following abbreviations are used in this manuscript:

EDs	Eating Disorders
AN	Anorexia Nervosa
BN	Bulimia Nervosa
BED	Binge Eating Disorder
LEDBs	Lifetime Eating Disorder Behaviors
ON	Orthorexia Nervisa

## References

[1] Micali N. (2022). What’s weighing us down: Closing the gap between the global burden of eating disorders and their representation. Eur. Child Adolesc. Psychiatry.

[2] Hay P., Aouad P., Le A., Marks P., Maloney D., Barakat S., Boakes R., Brennan L., Bryant E., Byrne S. (2023). Epidemiology of eating disorders: Population, prevalence, disease burden and quality of life informing public policy in Australia—A rapid review. J. Eat. Disord..

[3] Kiely L., Conti J., Hay P. (2023). Conceptualisation of severe and enduring anorexia nervosa: A qualitative meta-synthesis. BMC Psychiatry.

[4] Keel P.K., Brown T.A. (2010). Update on course and outcome in eating disorders. Int. J. Eat. Disord..

[5] Babb C., Jones C.R.G., Fox J.R.E. (2022). Investigating service users’ perspectives of eating disorder services: A meta-synthesis. Clin. Psychol. Psychother..

[6] Tragantzopoulou P. (2021). The long-lasting cycle of transformations in eating habits and the emergence of Orthorexia Nervosa: COVID-19 implications and future challenges. Sention J..

[7] Fairburn C.G., Cooper Z., Shafran R. (2003). Cognitive behavior therapy for eating disorders: A transdiagnostic theory and treatment. Behav. Res. Ther..

[8] Fairburn C.G., Cooper Z., Shafran R., Fairburn C.G. (2008). Enhanced cognitive behavior therapy for eating disorders: The core protocol. Cognitive Behavior Therapy and Eating Disorders.

[9] Fairburn C.G., Cooper Z., Doll H.A., O’Connor M.E., Bohn K., Hawker D.M., Wales J.A., Palmer R.L. (2009). Transdiagnostic cognitive-behavioral therapy for patients with eating disorders: A two-site trial with 60-week follow-up. Am. J. Psychiatry.

[10] Lampard A.M., Byrne S.M., McLean N., Fursland A. (2011). An evaluation of the enhanced cognitive-behavioural model of bulimia nervosa. Behav. Res. Ther..

[11] Lampard A.M., Tasca G.A., Balfour L., Bissada H. (2013). An evaluation of the transdiagnostic cognitive-behavioural model of eating disorders. Eur. Eat. Disord. Rev..

[12] Mares S.H.W., Burger J., Lemmens L.H.J.M., van Elburg A.A., Vroling M.S. (2022). Evaluation of the cognitive behavioural theory of eating disorders: A network analysis investigation. Eat. Behav..

[13] Meier M., McNally R.J., Kuckertz J.M., Braks K., Buhlmann U. (2025). A transdiagnostic model of eating disorders—Comparing symptom interactions in anorexia nervosa, bulimia nervosa, and binge eating disorder. Cogn. Ther. Res..

[14] Wade T.D., Bergin J.L., Martin N.G., Gillespie N.A., Fairburn C.G. (2006). A transdiagnostic approach to understanding eating disorders. J. Nerv. Ment. Dis..

[15] Prefit A.B., Cândea D.M., Szentagotai-Tătar A. (2019). Emotion regulation across eating pathology: A meta-analysis. Appetite.

[16] Belloli A., Saccaro L.F., Landi P., Spera M., Zappa M.A., Dell’Osso B., Rutigliano G. (2024). Emotion dysregulation links pathological eating styles and psychopathological traits in bariatric surgery candidates. Front. Psychiatry.

[17] Han S., Lee S. (2017). College student binge eating: Attachment, psychological needs satisfaction, and emotion regulation. J. Coll. Stud. Dev..

[18] Haynos A.F., Wang S.B., Fruzzetti A.E. (2018). Restrictive eating is associated with emotion regulation difficulties in a non-clinical sample. Eat. Disord..

[19] Breithaupt L., Rallis B., Mehlenbeck R., Kleiman E. (2016). Rumination and self-control interact to predict bulimic symptomatology in college students. Eat. Behav..

[20] Stice E. (1994). Review of the evidence for a sociocultural model of bulimia nervosa and an explanation of the mechanisms of action. Clin. Psychol. Rev..

[21] Stice E. (2001). A prospective test of the dual-pathway model of bulimic pathology: Mediating effects of dieting and negative affect. J. Abnorm. Psychol..

[22] Stice E., Rohde P., Shaw H. (2013). The Body Project: A Dissonance-Based Eating Disorder Prevention Intervention.

[23] Tragantzopoulou P., Mouratidis C., Paitaridou K., Giannouli V. (2024). The battle within: A qualitative meta-synthesis of the experience of the eating disorder voice. Healthcare.

[24] Olstad S., Solem S., Hjemdal O., Hagen R. (2015). Metacognition in eating disorders: Comparison of women with eating disorders, self-reported history of eating disorders or psychiatric problems, and healthy controls. Eat. Behav..

[25] Simonsen C.B., Jakobsen A.G., Grøntved S., Kjaersdam Telléus G. (2020). The mentalization profile in patients with eating disorders: A systematic review and meta-analysis. Nord. J. Psychiatry.

[26] Michopoulos V., Powers A., Moore C., Villarreal S., Ressler K.J., Bradley B. (2015). The mediating role of emotion dysregulation and depression on the relationship between childhood trauma exposure and emotional eating. Appetite.

[27] Moulton S.J., Newman E., Power K., Swanson V., Day K. (2015). Childhood trauma and eating psychopathology: A mediating role for dissociation and emotion dysregulation?. Child Abuse Negl..

[28] Martin S., Strodl E. (2023). The relationship between childhood trauma, eating behaviours, and the mediating role of metacognitive beliefs. Appetite.

[29] Dietitians Australia (2023). Eating Disorders Role Statement: What Dietitians Do in Eating Disorders. https://dietitiansaustralia.org.au/sites/default/files/2023-03/Eating-Disorder-Role-Statement-2023.pdf.

[30] Ozier A.D., Henry B.W. (2011). Position of the American Dietetic Association: Nutrition intervention in the treatment of eating disorders. J. Am. Diet. Assoc..

[31] Yang Y., Conti J., McMaster C.M., Hay P. (2021). Beyond refeeding: The effect of including a dietitian in eating disorder treatment. A systematic review. Nutrients.

[32] Heruc G., Hart S., Stiles G., Fleming K., Casey A., Sutherland F., Jeffrey S., Roberton M., Hurst K. (2020). ANZAED practice and training standards for dietitians providing eating disorder treatment. J. Eat. Disord..

[33] McMaster C.M., Wade T., Franklin J., Hart S. (2020). A review of treatment manuals for adults with an eating disorder: Nutrition content and consistency with current dietetic evidence. Eat. Weight Disord..

[34] Terry N., Reeves A. (2015). How do counsellors and psychotherapists understand diet and nutrition as part of the therapy process?. Couns. Psychother. Res..

[35] Hay P., Chinn D., Forbes D., Madden S., Newton R., Sugenor L., Touyz S., Ward W., Royal Australian and New Zealand College of Psychiatrists (2014). Royal Australian and New Zealand College of Psychiatrists clinical practice guidelines for the treatment of eating disorders. Aust. N. Z. J. Psychiatry.

[36] National Institute for Health and Care Excellence (NICE) (2020). Eating Disorders: Recognition and Treatment.

[37] Heruc G., Hurst K., Casey A., Fleming K., Freeman J., Fursland A., Hart S., Jeffrey S., Knight R., Roberton M. (2020). ANZAED eating disorder treatment principles and general clinical practice and training standards. J. Eat. Disord..

[38] Rosen M.A., DiazGranados D., Dietz A.S., Benishek L.E., Thompson D., Pronovost P.J., Weaver S.J. (2018). Teamwork in healthcare: Key discoveries enabling safer, high-quality care. Am. Psychol..

[39] Bray M., Heruc G., Wright O.R.L. (2025). From silos to synergy: A scoping review of team approaches to outpatient eating disorder treatment. Int. J. Eat. Disord..

[40] Elran-Barak R., Grundman-Shem Tov R., Zubery E., Lewis Y.D. (2024). Therapeutic alliance with psychotherapist versus dietician: A pilot study of eating disorder treatment in a multidisciplinary team during the COVID-19 pandemic. Front. Psychiatry.

[41] Brennan C., Baudinet J., Simic M., Eisler I. (2024). The role of the dietitian within family therapy for anorexia nervosa (FT-AN): A reflexive thematic analysis of child and adolescent eating disorder clinician perspectives. Nutrients.

[42] Denzin N.K., Lincoln Y.S. (2011). The Sage Handbook of Qualitative Research.

[43] Palinkas L.A., Horwitz S.M., Green C.A., Wisdom J.P., Duan N., Hoagwood K. (2015). Purposeful sampling for qualitative data collection and analysis in mixed method implementation research. Adm. Policy Ment. Health.

[44] Jamshed S. (2014). Qualitative research method—Interviewing and observation. J. Basic Clin. Pharm..

[45] Braun V., Clarke V. (2006). Using thematic analysis in psychology. Qual. Res. Psychol..

[46] Vervaet M., Puttevils L., Hoekstra R.H.A., Fried E., Vanderhasselt M.A. (2021). Transdiagnostic vulnerability factors in eating disorders: A network analysis. Eur. Eat. Disord. Rev..

[47] Tragantzopoulou P., Giannouli V. (2024). Unveiling anxiety factors in orthorexia nervosa: A qualitative exploration of fears and coping strategies. Healthcare.

[48] Fixsen A., Cheshire A., Tragantzopoulou P., von Kardorff E., Harbusch M., Robin D. (2025). Orthorexia Nervosa: The emergence of a psychiatric illness. Lay people and professionals’ constructions of extreme healthy eating. Zur Gesellschaft der Verletzten Seelen.

[49] Fixsen A., Ridge D., Cheshire A., Tragantzopoulou P. (2025). Paradoxical bio-citizenship: Examining healthy eating from lay and professional perspectives. Soc. Sci. Med..

[50] Di Lodovico L., Hamelin H., DeZorzi L., Tezenas du Montcel C., Schéle E., Stoltenborg I., Adan R., Dickson S., Gorwood P., Tolle V. (2024). What influences food choices in anorexia nervosa? Disentangling cognitive and emotional components of decision-making by translational research. Neurosci. Appl..

[51] Andreescu C.A., Pascual-Leone A., Nardone S. (2023). Disordered eating is related to deficits in emotional processing: A correlational study with a subclinical sample. J. Affect. Disord..

[52] Wells S.J. (2009). Transdiagnostic Features of Bulimia Nervosa and Binge Eating Disorder. Ph.D. Thesis.

[53] Adamidou K., Tragantzopoulou P. (2025). Opening Pandora’s Box: Exploring body image perceptions and influencing factors in women—An interpretative phenomenological analysis. Healthcare.

[54] Adamidou K., Tragantzopoulou P. (2025). “Seeing myself as a whole”: An IPA study exploring positive body image through Greek women’s embodied experiences. Women.

[55] Tragantzopoulou P. (2025). Hyper-visible yet invisible: Exploring the body image experiences of overweight women in everyday life. Obesities.

[56] Lindgreen P., Lomborg K., Clausen L. (2018). Patient experiences using a self-monitoring app in eating disorder treatment: Qualitative study. JMIR mHealth uHealth.

[57] Mueller-Stierlin A.S., Teasdale S.B., Dinc U., Moerkl S., Prinz N., Becker T., Kilian R. (2021). Feasibility and acceptability of photographic food record, food diary and weighed food record in people with serious mental illness. Nutrients.

[58] Hellner M., Steinberg D., Baker J., Cai K., Freestone D. (2024). Dietary interventions in family-based treatment for eating disorders: Results of a randomized comparative effectiveness study. Eat. Disord..

[59] Ellis A., Gillespie K., McCosker L., Hudson C., Diamond G., Machingura T., Branjerdporn G., Woerwag-Mehta S. (2024). Meal support intervention for eating disorders: A mixed-methods systematic review. J. Eat. Disord..

[60] Mack R.A., Kelleher K., Bhattarai J., Spence T. (2024). Individuals with eating disorders’ perspectives on a meal preparation intervention. Occup. Ther. Ment. Health.

[61] Bewell C.V., Carter J.C. (2008). Readiness to change mediates the impact of eating disorder symptomatology on treatment outcome in anorexia nervosa. Int. J. Eat. Disord..

[62] Lee H., Desai S., Choi Y.N. (2024). Improvements in quality of life and readiness for change after participating in an eating disorder psychoeducation group: A pilot study. Int. J. Group Psychother..

[63] Prochaska J.O., DiClemente C.C., Norcross J.C. (1992). In search of how people change: Applications to addictive behaviors. Am. Psychol..

[64] Norcross J.C., Krebs P.M., Prochaska J.O. (2011). Stages of change. J. Clin. Psychol..

[65] Leclerc A., Turrini T., Sherwood K., Katzman D.K. (2013). Evaluation of a nutrition rehabilitation protocol in hospitalized adolescents with restrictive eating disorders. J. Adolesc. Health.

[66] Marzola E., Nasser J.A., Hashim S.A., Shih P.B., Kaye W.H. (2013). Nutritional rehabilitation in anorexia nervosa: Review of the literature and implications for treatment. BMC Psychiatry.

[67] Chaudhary A., Sudzina F., Mikkelsen B.E. (2020). Promoting healthy eating among young people—A review of the evidence of the impact of school-based interventions. Nutrients.

[68] Marchionatti L.E., Schafer J.L., Karagiorga V.E., Balikou P., Mitropoulou A., Serdari A., Moschos G., Athanasopoulou L., Basta M., Simioni A. (2024). The mental health care system for children and adolescents in Greece: A review and structure assessment. Front. Health Serv..

[69] Novogrudsky K., Gray T., Mitchell E., Attoe C., Kern N., Griffiths J., Serpell L., Treasure J., Schmidt U. (2025). A novel whole-team training programme for adult eating disorder services in England: Rationale, development and preliminary evaluation. BJPsych Bull..

[70] Janicic A., Bairaktari M. (2014). Eating disorders: A report on the case of Greece. Adv. Eat. Disord..

[71] Tragantzopoulou P., Giannouli V., Filippou A., Demirtzidou M. (2024). Burnout and coping strategies in integrative psychotherapists: Findings from qualitative interviews. Healthcare.

[72] Tragantzopoulou P., Giannouli V. (2020). Eating disorders and body image disturbance among males and females: From the perspective of six therapists with different therapeutic orientation. Anthropol. Res. Stud..

[73] Tragantzopoulou P., Giannouli V. (2023). “You feel that you are stepping into a different world”: Vulnerability and biases in the treatment of anorexia nervosa. Eur. J. Psychother. Couns..

[74] Tsamalidou A., Tragantzopoulou P. (2025). From doubt to development: Professional journeys of novice CBT therapists. Behav. Sci..

[75] Demirtzidou M., Tragantzopoulou P. (2025). Mitigating burnout: A qualitative exploration of clinical supervision’s impact on novice psychotherapists. Eur. J. Psychother. Couns..

[76] Tragantzopoulou P., Giannouli V. (2025). Seeking support and treatment: A thematic analysis of tweets about the experience of endometriosis. J. Endometr. Pelvic Pain Disord..

[77] Tragantzopoulou P., Fixsen A., Ridge D., Cheshire A. (2024). ‘You are not alone, we’ve got you’: Power plays, devotion, and punishment on healthy eating and pro–eating disorder websites. Qual. Health Res..

